# Dr. A. S. Payne

**Published:** 1875-03

**Authors:** 


*Dr. A. S. Payne.*

We desire to call the attention of our readers to the very learned and interesting article in this issue from the pen of our Virginia associate, Dr. Payne. While the article is longer than we usually publish entire—remembering always our motto, "*Quicquid Præcipes esto Brevis*"—yet we do so in this instance with no hesitancy, but with great pleasure, knowing it will be read by our readers with real gratification and profit. It affords us pleasure in this connection to say to Dr. Tuner and his friends of Tennessee, that the request that some one of our contributors should furnish an article giving the history and treatment of typhoid pneumonia (and typhoid fever), will be ably undertaken by Dr. Payne, which in due time will appear in this journal. Of course those of our readers who know Dr. Payne may expect something interesting, new and profitable.

